# Epidemiological Features and Clinical Manifestations of Patients With Somatoform Disorder at a Tertiary Medical City in Riyadh, Saudi Arabia

**DOI:** 10.7759/cureus.19799

**Published:** 2021-11-22

**Authors:** Abdulkarim O Alanazi, Raed A Aljohani, Mohammad F Aljohani, Abdulmohsen A Alhussaini, Faisal K Alnemer, Salman S Qasim, Ghada S Alduraye, Laila Layqah, Fares F Alharbi

**Affiliations:** 1 College of Medicine, King Saud Bin Abdulaziz University for Health Sciences, Riyadh, SAU; 2 Department of Mental Health, Ministry of the National Guard Health Affairs, Riyadh, SAU; 3 Department of Research Office, King Abdullah International Medical Research Center, Riyadh, SAU

**Keywords:** somatoform disorder, somatization disorder, conversion disorder, pain disorder, hypochondriasis, saudi arabia

## Abstract

Background

Somatoform disorder (SD), known as the presence of physical symptoms suggesting a physical condition, for which there are no demonstrable organic findings or established physiological mechanisms with positive evidence that the symptoms are related to psychological causes. The aim of this study was to highlight the epidemiological characteristics, demographic features, comorbidities, and clinical presentations of patients with SD.

Materials and methods

This was a retrospective study of SD patients at King Abdulaziz Medical City in Riyadh, Saudi Arabia. We reviewed the patients' electronic health records from January 2015 to December 2020 for collecting the patients’ demographic information, including gender, age, marital status, and occupation. The types of SD, presenting symptoms of each disorder, department to which patients initially presented, comorbidities, and management were also documented. The diagnosis of SD was based on the International Classification of Diseases, Tenth Revision (ICD-10).

Results

In total, 89 patients were included in the study. The majority (n=50, 56.2%) were female, with a mean age of 42.7±17.1 years. More than half of the sample was married (n=54, 60.7%). The most common subtype of SD was somatization disorder followed by conversion disorder, pain disorder, and hypochondriasis, diagnosed in 69 (77.5%), 12 (13.5%), 5 (5.6%), and three (3.4%) patients, respectively. Neurological symptoms and pain were the most frequent presenting symptoms for all the somatoform patients. More than half of the sample (n=48, 53.9%) initially presented at an outpatient clinic.

Conclusions

The number of SD patients was less than expected, and a third did not receive any treatment. This emphasizes the need for more SD awareness among clinicians in various medical specialties. Appropriate SD and other mental disorders education for physicians may support achieving a better identification of SD and subsequently an improved quality of life for the patients.

## Introduction

The presence of physical symptoms that are suggestive of a medical condition for which there are no demonstrable organic findings or established physiological mechanisms, with evidence that the symptoms are related to psychological causes, is known as somatoform disorder (SD) [1]. Somatization, conversion disorder, hypochondriasis, and pain disorder are some of the SD included in the taxonomy of the International Classification of Diseases, Tenth Revision (ICD-10). SD is distinguished by frequently changing physical symptoms that cannot be explained by any existing medical condition [2]. SD is not infrequent in communities and is seen in primary health care centers [3]. According to a systemic review involving multiple studies conducted in 24 different countries, the prevalence of SD ranges from 26.2% to 34.8% among primary care patients [4]. A retrospective study conducted by Ng et al. [5] in psychiatric outpatient clinics in a rural area of the United States of America (USA), indicated an SD prevalence of 5%, and a survey conducted in Los Angeles, USA found an SD prevalence of 4.4% in the community [6]. The prevalence of SD in Europe ranges from 1.1% to 11.0%, according to six community surveys [7]. In terms of the Middle East, a study done by Bener et al. in Qatar reported a prevalence of 12.4% in 1,689 patients attending primary care centers [8]. Another cross-sectional survey-based study conducted by Mahmoud et al. in the United Arab Emirates (UAE) reported a 4% prevalence in 530 patients among the city’s adult residents [9]. Local studies in Saudi Arabia considering the prevalence of SD are lacking, however, a study done by Alqahtani et al. in the Asir region reported a prevalence of 16% in primary healthcare centers [10].

SD is prevalent and is challenging for physicians to diagnose and treat. Instead of presenting with psychological complaints, many SD patients present with unexplained physical symptoms, which results in an overabundance of expensive clinical investigations. This disorder is underdiagnosed, physicians frequently explore an organic explanation for their patients' concerns using many medications, diagnostic and surgical procedures, rather than diagnosing SD [8]. Due to a lack of local studies assessing the epidemiological characteristics of patients with SD, we aimed in the present study to evaluate demographic features, comorbidities, and clinical presentations of patients with SD at a major referral medical city in Riyadh, Saudi Arabia. The findings of this study may support achieving a better identification of SD and subsequently improved medical care and quality of life for the patients.

## Materials and methods

This was a retrospective study, including patients diagnosed with SD and attending King Abdulaziz Medical City (KAMC) in Riyadh, Saudi Arabia. National Guard soldiers and their families receive healthcare at KAMC, with a capacity of 1,501 beds. Healthcare facilities at KAMC include medical and surgical wards, the psychiatric department, antenatal and postpartum wards, intensive care units, long-term care and rehabilitation wards, trauma and emergency departments, ambulatory and dental clinics, and obstetrics and gynecology department. The medical city also includes King Abdullah Specialist Children's Hospital, which is Saudi Arabia's main referral center for pediatric patients, as well as a specialized cardiac center. We reviewed the patients' electronic health records from January 2015 to December 2020, using the BESTCare health information system, which was introduced at our institution in 2015. The inclusion criterion was all patients who were diagnosed with SD during that period. The diagnosis of SD by psychiatrists was based on ICD-10.

Demographic information, including gender, age, marital status, and occupation, was extracted from the electronic health records. The types of SD, presenting symptoms of each disorder, department to which patients initially presented, comorbidities, and management were also documented.

The present study was approved by the Institutional Review Board of King Abdullah International Medical Research Center, Ministry of National Guard-Health Affairs, Riyadh, Saudi Arabia (approval number RC20/607/R), prior to data collection. Patient confidentiality was ensured, and the data were only received and used by the research team. The patient’s medical record number was replaced with a serial number and saved in a separate sheet accessible only to the primary investigator to ensure confidentiality.

## Results

Table 1 displays the baseline characteristics of the included in the study period sample (n=89). Out of the whole sample, the majority (56.2%) were females, with a mean age of 42.7±17 years. Most of the patients were married (60.7%) and 40% were unemployed (Table 1).

**Table 1:** Demographic characteristics of the sample (n=89)

Demographic characteristic	Category	N (%)
Gender	Females	50 (56.2)
	Males	39 (43.8)
Age (years)	<25	14 (15.7)
	25-44	38 (42.7)
	45-65	29 (32.6)
	>65	8 (9)
Marital status	Married	54 (60.7)
	Single	30 (33.7)
	Widowed	3 (3.4)
	Divorced	2 (2.2)
Occupation	Employed	27 (30.3)
	Unemployed	36 (40.4)
	Student	13 (14.6)
	Retired	13 (14.6)

Table 2 shows the presenting symptoms of patients diagnosed with somatization disorder, and Table 3 displays the other types of SD with their presenting symptoms. Some patients presented with two or more symptoms. The types of SD were somatization disorder, conversion disorder, hypochondriasis, and pain disorder, with the most prevalent being somatization disorder (n=69, 77.5%), followed by conversion disorder (n=12, 13.5%). Pain symptoms were the most frequent presenting symptom in the somatization group (n=39, 56.2%), followed by neurological symptoms (n=32, 46.4%), gastrointestinal symptoms (n=7, 10.1%), other symptoms such as fatigue, insomnia, and cough (n=7, 10.1%), and cardiovascular symptoms (n=6, 8.7%). Regarding the conversion disorder group, all patients presented with neurological symptoms. One patient (8.3%) presented with generalized pain, one (8.3%) with syncope, and one (8.3%) with nausea. All patients in the pain disorder group presented with pain symptoms. Moreover, one patient (20%) presented with cough, and one (20%) with shortness of breath. Lastly, the three patients in the hypochondriasis group presented with different symptoms, one (33.3%) with chest pain, one (33.3%) with a skin lesion and rashes, and one (33.3%) with nausea. Additional explanation of each subcategory is shown in Table 2 and Table 3.

**Table 2:** Presenting symptoms of patients with somatization disorder

Somatization disorder (n=69, 77.5%)	N (%)
Pain symptoms	39 (56.2)
Headache	14 (20.3)
General body pain	10 (14.5)
Abdominal pain	6 (8.7)
Chest pain	5 (7.2)
Shoulder pain	4 (5.8)
Joint pain	4 (5.8)
Ear pain	3 (4.3)
Epigastric pain	3 (4.3)
Neck pain	3 (4.3)
Back pain	2 (2.9)
Knee pain	2 (2.9)
Leg pain	1 (1.4)
Facial pain	1 (1.4)
Gastrointestinal symptoms	7 (10.1)
Vomiting	4 (5.8)
Nausea	3 (4.3)
Diarrhea	3 (4.3)
Constipation	2 (2.9)
Loss of appetite	1 (1.4)
Neurological symptoms	32 (46.4)
Left side weakness	7 (10.1)
Dizziness	6 (8.7)
Numbness	5 (7.2)
Loss of consciousness	4 (5.8)
Right side weakness	3 (4.3)
Facial weakness	3 (4.3)
Tremor	3 (4.3)
Blindness	2 (2.9)
Confusion	1 (1.4)
Generalized weakness	1 (1.4)
Feet hotness	1 (1.4)
Tinnitus	1 (1.4)
Slurred speech	1 (1.4)
Cardiovascular symptoms	6 (8.7)
Shortness of breath	3 (4.3)
Palpitation	3 (4.3)
Syncope	1 (1.4)
Others	7 (10.1)
Fatigue	6 (8.7)
Cough	2 (2.9)
Insomnia	1 (1.4)

**Table 3:** Presenting symptoms of patients with conversion disorder, pain disorder, and hypochondriasis

Conversion disorder (n= 12, 13.5%)	N (%)
Left side weakness	7 (58.3)
Numbness	4 (33.4)
Slurred speech	4 (33.4)
Facial weakness	3 (25)
Generalized weakness	2 (16.7)
Sensory neural hearing loss	1 (8.3)
Blindness	1 (8.3)
Right side weakness	1 (8.3)
Dizziness	1 (8.3)
General body pain	1 (8.3)
Syncope	1 (8.3)
Nausea	1 (8.3)
Pain disorder (n=5, 5.6%)	N (%)
Abdominal pain	1 (20)
Chest pain	1 (20)
Back pain	1 (20)
Leg pain	1 (20)
Foot pain	1 (20)
Cough	1 (20)
Shortness of breath	1 (20)
Hypochondriasis (n=3, 3.4%)	N (%)
Chest pain	1 (33.3)
Skin lesions and rashes	1 (33.3)
Nausea	1 (33.3)

Additional analysis indicated that more than half of the sample (n=48, 53.9%) initially presented to an outpatient clinic, with 41 (46.1%) presenting to the emergency department (ED). The most frequent presenting symptoms of all the somatoform groups were pain (n=46, 51.7%) and neurological symptoms (n=44, 49.4%), followed by gastrointestinal, cardiovascular, and other symptoms. Regarding comorbidities, hypertension was reported in 22 (24.7%), followed by diabetes mellitus in 15 (16.9%), and dyslipidemia in 12 (13.5%) (Table 4).

**Table 4:** Presenting department, symptoms, and comorbidities BMI: body mass index

Variable	Outcome	N (%)
Presenting department	Outpatient clinic	48 (54)
	Emergency department	41 (46)
Comorbidities	Hypertension	22 (24.7)
	Diabetes mellitus	15 (16.9)
	Dyslipidemia	12 (13.5)
	Asthma	8 (9)
	Ischemic heart disease	7 (7.9)
	Chronic kidney disease	3 (3.4)
	Obesity (BMI > 30)	15 (16.9)
	Smoking	7 (7.9)
Symptoms	Pain symptoms	46 (51.7)
	Neurological symptoms	44 (49.4)
	Gastrointestinal symptoms	9 (10)
	Cardiovascular symptoms	8 (9)
	Others	8 (9)

The pharmacological treatment of SD included antidepressants (n=41, 46%), anticonvulsants (n=15, 16.9%), atypical antipsychotics (n=10, 11.2%), benzodiazepines (n=6, 6.7%), non-steroidal anti-inflammatory drugs (NSAIDs) (n=1, 1.1%), and paracetamol (n=1, 1.1%). However, more than one-third (n=37, 41.6%) did not receive any pharmacological treatment. Cognitive behavioral therapy was only offered to three (3.4%) patients.

## Discussion

In the present study, we found that most of the sample was in the 25-44 year age group followed by the 45-65-year age group. This result is similar to several studies done on SD [1,3,10-11]. In terms of gender distribution, more than half of the patients (56.2%) were females. Likewise, Rief et al. [3] reported a female prevalence of 63.2% in the general population of Germany, and a community survey conducted in Florence, Italy, reported a female prevalence of 54.8% [1]. Moreover, 75% of patients presenting to primary care clinics in the United Kingdom with unexplained physical symptoms were found to be females [12]. Other reports from Middle Eastern countries also indicated a female predominance of SD [8-10]. This difference in gender distribution may be attributed to several factors. Men, especially in Middle Eastern countries, are perceived to be less expressive about their illness and distress [13]. This is to preserve their sense of masculinity as their societies define it. Denying illness, weakness, or distress may subsequently result in fewer men presenting to the clinic with symptoms related to SD. Men and women may also remember previous medical experiences differently, with men forgetting their symptoms more easily than women [13-15], resulting in a recall bias.

In the present study, 60.7% were married, 3.4% were widowed, and 2.2% were divorced. A study conducted in China found that more than 80% of the patients with SD were married, and only 14.4% were single [16]. Farvelli et al. [1] reported that 70% of the sample were married, and 19.6% were single. Bener et al. [8] found a similar result, with 85.2% married and 14.8% single, which is in line with our results. We believe that a possible explanation for this may be related to the burden of domestic responsibilities, affecting the mental health of both the husband and wife. Partners undergo various degrees of stress during different stages of marriage. The husband or wife may abstain from expressing their feelings of disappointment or upset in order to save their marriage. Those suppressed feelings may appear as physical symptoms if stressors were not effectively managed.

As SD is classified into several categories, each patient has a unique clinical presentation. In the present study, the majority were diagnosed with somatization disorder. This is in agreement with Farvelli et al. [1] and Fink et al. [17]. The proportions of the other categories in our study were low compared to somatization disorder, which could be due to a lack of awareness of these categories among non-psychiatric physicians, and the small sample size.

In the current study, obesity was present in 15 (16.9%) patients. A study conducted in the general population in Lubeck, Germany, with a sample size of 4033 after excluding pregnant women and patients with a coexisting eating disorder, indicated that obesity has no relation with SD [18]. In contrast, a population-based study done in Germany found a high rate of somatoform and eating disorders identified in a clinical sample of obese adolescents, higher than the population controls [19]. Increased rates of physical health problems and depression are two possible reasons for the association between obesity and somatic symptoms [20]. This disparity in the literature may be attributed to the difference in the population's age groups.

Presenting symptoms of patients diagnosed with somatization disorder can vary widely. Pain is believed to be the most commonly reported symptom among patients with somatization disorder [21]. Likewise, patients who were diagnosed with somatization disorder in this study presented with pain. The pain was reported in various areas of the body, such as the abdomen, chest, joints, and ears, with headache being the most prevalent form of pain, evident in half of the sample. In a cross-sectional Nigerian survey with 60 SD patients, Obimakinde et al. [22] reported that 88.3% of the patients presented with headache. Generalized body pains (35.9%) were the second most frequent presenting symptoms, a slightly higher figure than what was reported by Obimakinde et al. [22]. The specific pattern of somatization varies according to the ethnic/racial background of the somatizer. In black Africans and South Asian countries, for instance, sensations of worms in the head or ants crawling under the skin are prevalent [23]. This symptom may be frequent in Africans, potentially due to the interaction with catastrophic thinking and the cultural resemblance of this idiomatic manifestation of psychic distress [22-23].

Symptoms of conversion disorder are believed to be severe enough to cause impairment, affecting movement, function, and sense, and warranting prompt medical attention [24]. All of the patients diagnosed with conversion disorder in the present study presented with neurological symptoms, with left-sided weakness being the most common. This is consistent with the notion that weakness or paralysis in conversion disorder patients is usually confined to one-half of the body and does not follow any specific anatomical pattern [25]. The second most commonly reported neurological symptoms among patients with conversion disorder were numbness and slurred speech, which were found in approximately one-third of the patients. In the literature, functional dysphonia (hoarseness or whispering) was the most commonly reported speech symptom among conversion disorder patients [25]. It is worth noting that conversion disorder patients with functional dysphonia have a normal vocal cords examination and are able to cough or sing, unlike patients with true dysphonia who have difficulty doing so [25]. It is not unexpected that patients with conversion disorder present with at least one neurological symptom that affects body movement or sense, as it constitutes a criterion for its diagnosis in The Diagnostic and Statistical Manual of Mental Disorders, fifth edition (DSM-5) [23]. Those symptoms must not be explainable by any other neurological or medical condition in order to fulfill the diagnostic criteria [23,25].

In terms of a pain disorder, the patients may present with pain in different sites in the body. A general population survey done in Germany reported lower back pain with the highest prevalence in both genders, followed by headache and joint pain [26]. Farvelli et al. found that pain disorder patients present with back pain, pain in the extremities, and pain in other sites, 50% for each site [1]. In the present study, the patients presented with either abdominal pain, chest pain, back pain, leg pain, or foot pain, 20% for each symptom, which is lower than the previous study. The least prevalent disorder in this study was hypochondriasis with a prevalence of 3.4%. The presenting symptoms were abdominal pain, chest pain, skin lesions, and nausea, 33.3% for each symptom, which is also supported by Farvelli et al. [1].

SD patients are usually treated through pharmacological and non-pharmacological treatment. Anti-depressants, antipsychotics, and anticonvulsants are common drugs used to treat somatic symptoms [27]. In the present research, 46% were treated pharmacologically with an anti-depressant, 16.9% treated with an anticonvulsant, 11.2% with an atypical antipsychotic, and with other drugs such as benzodiazepine, NSAIDs, and paracetamol. This is similar to studies in psychiatric hospitals in Germany, Austria, Switzerland, Hungary, and Belgium done by Mundt et al. [28], who reported that the majority of the patients were prescribed an anti-depressant. This could be due to a preference over the other classes of medication, because of the adverse effects profile. Selective serotonin reuptake inhibitors were less prominent, which improves compliance [29]. However, in the present study, the second most used drug was an anti-convulsant, unlike Mundt et al. [28] in which the second most used drugs were neuroleptics, now known as anti-psychotic. This could be due to the absence of clear-cut pharmacological recommendations. A variety of psychotropic and somatic substances is currently used for treatment in clinical practice. Regarding psychotherapy, a systemic review of 29 randomized studies reported that of 1689 patients, 47.5% received CBT [30]. Unlike the previous study, only 3.4% of the patients in our study received CBT.

The limitations of the current study were the small sample size, the retrospective design, and the fact the data was only collected from one center; possibly limiting the generalizability of the findings. This topic requires more research to be done in Saudi Arabia, with a larger sample size to achieve an improved outcome for this disorder at the local and international levels.

## Conclusions

The number of SD patients in the present study is likely underestimated and is expected to be higher. About a third of the patients did not receive any medical treatment. This emphasizes the need for more SD awareness among clinicians in various medical specialties, which can be achieved through the implementation of in-service training of clinicians from different disciplines and raising awareness of SD in medical school education by encouraging academic teaching on SD. Appropriate SD and other mental disorders education for physicians may support achieving a better identification of SD and subsequently an improved quality of life for the patients.
